# Dietary Anthocyanins as Nutritional Therapy for Nonalcoholic Fatty Liver Disease

**DOI:** 10.1155/2013/145421

**Published:** 2013-10-24

**Authors:** Luca Valenti, Patrizia Riso, Alessandra Mazzocchi, Marisa Porrini, Silvia Fargion, Carlo Agostoni

**Affiliations:** ^1^Department of Pathophysiology and Transplantation (DEPT), Università degli Studi di Milano, Internal Medicine, Fondazione IRCCS Ca' Granda Ospedale Policlinico, 20122 Milano, Italy; ^2^Division of Human Nutrition, Department of Food, Environmental and Nutritional Sciences (DeFENS), Università degli Studi di Milano, 20122 Milano, Italy; ^3^Pediatric Clinic 2, Department of Clinical Sciences and Community Health, Università degli Studi di Milano, Fondazione IRCCS Ca' Granda Ospedale Policlinico, 20122 Milano, Italy

## Abstract

Nonalcoholic fatty liver disease (NAFLD), defined by excessive lipid accumulation in the liver, is the hepatic manifestation of insulin resistance and the metabolic syndrome. Due to the epidemics of obesity, NAFLD is rapidly becoming the leading cause of altered liver enzymes in Western countries. NAFLD encompasses a wide spectrum of liver disease ranging from simple uncomplicated steatosis, to steatohepatitis, cirrhosis, and hepatocellular carcinoma. Diet may affect the development of NAFLD either by increasing risk or by providing protective factors. Therefore, it is important to investigate the role of foods and/or food bioactives on the metabolic processes involved in steatohepatitis for preventive strategies. It has been reported that anthocyanins (ACNs) decrease hepatic lipid accumulation and may counteract oxidative stress and hepatic inflammation, but their impact on NAFLD has yet to be fully determined. ACNs are water-soluble bioactive compounds of the polyphenol class present in many vegetable products. Here, we summarize the evidence evaluating the mechanisms of action of ACNs on hepatic lipid metabolism in different experimental setting: *in vitro*, *in vivo*, and in human trials. Finally, a working model depicting the possible mechanisms underpinning the beneficial effects of ACNs in NAFLD is proposed, based on the available literature.

## 1. Introduction

In the last decades, the pandemic of overweight and obesity related to sedentary lifestyle and excess intake of refined foods has led to a dramatic rise in the prevalence of the metabolic syndrome and associated conditions, such as type 2 diabetes and dyslipidemia, leading to accelerated atherosclerosis [1], but also to nonalcoholic fatty liver disease (NAFLD) [2, 3].

Lifestyle and dietary habits represent both major risk and protective factors in the development and progression of degenerative diseases [4].

Diets rich in fruits and vegetables are among the recommended lifestyle modifications to decrease the risk of degenerative diseases, such as cardiovascular disease but also to reduce the complications associated with metabolic disorders and advanced atherosclerosis. Diet is in fact affordable and available and usually does not include the side effects and the metabolic and physiologic burden that medications impose on body systems [5].

In this regard, many different dietary components are under study for their possible pharmacologic activity in several pathophysiological conditions at different levels (e.g., vascular, immune, hepatic, etc.).

Most bioactive compounds have been documented in fruits and vegetables [6] and their mechanisms of action investigated both in *in vitro* and in *in vivo* models. In particular, great interest has been devoted to several classes of polyphenols and especially to a specific subset of molecules called anthocyanins (ACNs).

## 2. Anthocyanins

**Figure 1:**
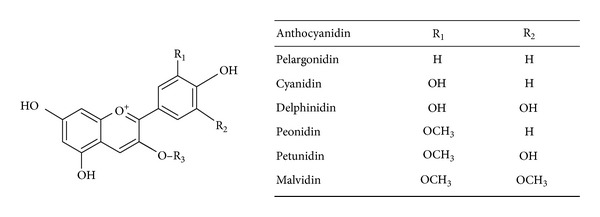
General chemical structures of anthocyanins in the diet. R_3_ = sugar (i.e., glucose, arabinose, galactose, as monomers, or dimers). Sugars can be present also on ring A; moreover acylation of sugars with aliphatic and/or aromatic acids can be found.

ACNs are water-soluble bioactive compounds, which belong to the widespread group named flavonoids within the polyphenol class. Chemically, ACNs consist of two aromatic rings linked by three carbons in an oxygenated heterocycle. The chromophore of ACNs is the 7-hydroxyflavylium ion. In particular, ACNs consist of an aglycon base or flavylium ring (anthocyanidins), sugars, and possibly acylating groups (Figure 1) [7]. ACNs are responsible for the red, purple and blue colors of many flowers, cereal grains, fruit, and vegetable. They are generally found in the skins, and their content is usually proportional to color intensity. ACN content varies greatly depending on the different food sources considered (Table 1) [8]. More than 600 different ACNs have been identified in vegetables, derived from twenty-three different aglycones (anthocyanidins) classified according to the number and position of hydroxyl and methoxyl groups on the flavan nucleus. The six anthocyanidins commonly found in fruit and vegetables are pelargonidin, cyanidin, delphinidin, peonidin, petunidin, and malvidin which are combined with sugars (mostly glucose, galactose, and arabinose) (Figure 1) [8].

ACN intake has been estimated to range between 180 mg/day and 215 mg/day, but these values can be 10 times lower in industrialized countries [9–11]. ACN bioavailability is reported to be lower than that of other polyphenols, and less than 1% of consumed ACNs is generally absorbed, reaching plasma concentrations in the nanomolar order [12]. In addition, ACNs are rapidly metabolized and their presence in the circulation is limited to a few hours. Despite their low absorption and rapid metabolism, the regular intake of ACNs may result in beneficial effects on human health by reducing the risks of cardiovascular disease and cancer [13–15]. Indeed, they possess high antioxidant capacity and can play a key role in the prevention of oxidative stress by scavenging reactive oxygen species and free radicals and by modulating endogenous defense system, as demonstrated in several *in vitro* and *in vivo* studies [16–18]. ACNs have also been documented to ameliorate hyperglycemia, to modulate endothelial function, and to decrease inflammation [19–24]. Moreover recently ACNs have been studied for their role in the modulation of lipid metabolism and fat deposition [25–27] in different tissues, including the liver.

## 3. Nonalcoholic Fatty Liver Disease

NAFLD is characterized by liver fat deposition, that is, steatosis, related to systemic insulin resistance (IR) [28]. In susceptible individuals, steatosis may be associated with oxidative hepatocellular damage, inflammation, and activation of fibrogenesis, defining nonalcoholic steatohepatitis (NASH) [29, 30]. NASH, but not simple steatosis, is a potentially progressive liver disease leading to cirrhosis and hepatocellular carcinoma [31]. Following the epidemics of obesity and the metabolic syndrome, NAFLD is rapidly becoming the leading cause of altered liver enzymes in Western countries [2, 32, 33], and NASH will become the major cause of end-stage liver disease and hepatocellular carcinoma within the next 10–20 years.

Fatty liver, that is, hepatic fat accumulation exceeding 5% of total liver mass, results from an unbalance between triglyceride deposition and synthesis on one hand and oxidation and secretion by lipoproteins on the other hand [34] and initially represents a protective mechanism against the toxicity resulting from an increased flux of free fatty acids (FFAs) to the liver [35]. Most of excess hepatic lipid content derives from increased peripheral lipolysis [36], which is caused by adipose tissue insulin resistance [37], and is a typical feature of obesity. Other contributing factors are increased lipogenesis induced by hyperinsulinemia or directly by diet. Indeed, the major risk factor for NAFLD is systemic IR due to central obesity and the metabolic syndrome [28, 38]. Impaired ability to secrete lipoproteins [39] and changes in fattyacid oxidation also contribute to hepatic fat accumulation [40].

Development of NASH has classically been explained by the occurrence of a so-called second-hit, leading to the activation of inflammation, in the context of hepatic steatosis (the “first hit”) [41]. This second insult likely represents a combination of insults related to (a) direct hepatic lipotoxicity, (b) hepatocellular oxidative stress secondary to free radicals produced during *β*- and *ω*-oxidation of FFAs, (c) inflammation triggered by endotoxins engaging Toll-like receptor-4 (TLR-4) in Kupffer cells (the hepatic macrophages) and hepatocytes due to increased intestinal permeability, bacterial overgrowth, and altered intestinal flora [42–44], (d) cytokine release, and (e) endoplasmic reticulum stress. These combine to produce inflammation, cellular damage, and activation of fibrogenesis. Genetic factors, and in particular the I148 M variant of Patatin-like phospholipase domain containing-3 (PNPLA3), play a major role in determining individual susceptibility to develop steatosis or NASH and progressive liver disease, interacting with dietary factors [45, 46].

## 4. Anthocyanins in NAFLD

Recent studies documented that ACNs can reduce hepatic lipid accumulation, but their impact on NAFLD has yet to be determined.

We have classified the available evidence according to the experimental setting: *in vitro*, *in vivo*, and in human trials. For the revision of the literature, the PubMed database was searched up to June 2013 (keywords: steatosis or nonalcoholic fatty liver disease or steatohepatitis plus anthocyanins or single anthocyanin names). No publication data restrictions were applied. Papers were selected for inclusion in this review on the basis of their relevance, and additional papers were obtained from their reference lists.

### 4.1. *In Vitro*

Studies evaluating the effect of ACNs *in vitro* on lipid metabolism and oxidative stress in hepatocytes, typical of NAFLD and NASH, are presented in Table 2. Most studies were conducted in human hepatoma HepG2 cells [47–55], an established model of hepatic lipid metabolism. Both ACN-rich extracts of foods (berries and potatoes) and synthetic ACNs (cyanidin hydrochloride and cyanidin-3-O-*β*-glucoside) were employed. Unfortunately, interpretation of the overall evidence is hindered by differences in cellular models, experimental protocols, and the molecular pathways evaluated. However, most studies are concordant on the fact that ACNs reduce hepatocellular lipid accumulation [48–50, 53–55] by inhibiting lipogenesis [49] and possibly by promoting lipolysis [53–55], although the different aspects of lipid metabolism were not evaluated in all studies. Furthermore, ACNs also reduce cellular oxidative stress by promoting the antioxidant response [47, 51, 52]. Interestingly, three independent studies reported that activation of the adenosine monophosphate protein kinase (AMPK) pathway was implicated in mediating the effect of ACNs on hepatic lipid metabolism and antioxidant response [49, 51, 53, 54]. However, another study suggested that ACNs may act as direct agonist of PPAR receptors in hepatocytes [55].

### 4.2. *In Vivo*

Studies evaluating the effect of ACNs *in vivo* on hepatic lipid metabolism, steatosis, oxidative stress, and steatohepatitis are presented in Table 3. Also in this case, the interpretation of the overall evidence is difficult, due to the very different experimental models of NAFLD and metabolic syndrome employed and to the different outcomes for the evaluation of lipid metabolism, oxidative stress, and liver damage. In addition, in some studies, animals were exposed to synthetic ACNs (i.e., cyanidin-3-O-*β*-glucoside) [50, 52, 56, 57], whereas in others they were exposed to extracts of ACN-rich foods (e.g., sweet potato, berries, and oranges) [27, 49, 58–62]. Mirroring the results obtained *in vitro*, there is ample convergence supporting an effect of ACNs in reducing hepatic lipid accumulation, that is, steatosis [49, 50, 52, 56–58, 60–63]. In addition, the majority of studies also reported an improvement in hepatic and systemic IR and serum lipids, often related to reduced weight gain [57, 58, 60–62]. Again, increased activation of PPAR*α* inducing lipolysis and reduced lipogenesis were postulated to be responsible for decreased hepatic fat content [27, 59–61]. Increased activity of the AMPK pathway was confirmed *in vivo* in one study [49], and increased hepatic antioxidant activity after exposure to ACN was also widely confirmed in experimental models of NAFLD [52, 56, 59, 63, 64]. However, whether improved redox status was secondary to or independent of reduced hepatic lipids and improved metabolic status was not tested. In some studies, these effects of ACN exposure translated in an improvement in inflammation, that is, in reduced severity of steatohepatitis [53, 58, 60]. The involvement of AMPK activation in mediating the beneficial effect of ACN on insulin sensitivity is also supported by evidence that bilberry extract ameliorates insulin resistance and hepatic lipid metabolism via this pathway [65].

### 4.3. Clinical Studies

There is only one study evaluating the effect of ACN on NAFLD patients, which is summarized in Table 4 [66]. Suda and coworkers recruited 48 adult men with increased liver enzymes negative for viral hepatitis, thereby likely affected by NAFLD. During a eight-week intervention, about 200 mg of acylated ACNs or placebo was administered twice daily. Acylated ACN intake was associated with reduced levels of liver enzymes, in particular gamma-glutamyltransferases. However, liver damage was not directly assessed, fatty liver was not confirmed by direct imaging, and the effect of acylated ACNs was not compared to that of a control food or to the lack of intervention.

## 5. Conclusions

It is widely accepted that exploring the role of foods and more specifically the effect of bioactive compounds such as ACNs on the metabolic processes involved in *chronic diseases* is critical for preventive strategies. For instance, similar therapeutic activities have been shown for docosahexaenoic acid on steatosis severity in children with NAFLD [67]. The availability of data demonstrating cause-effect relationships and the specific mode of action of such compounds are of paramount importance in order to support any dietary recommendation or supplementation.

**Figure 2:**
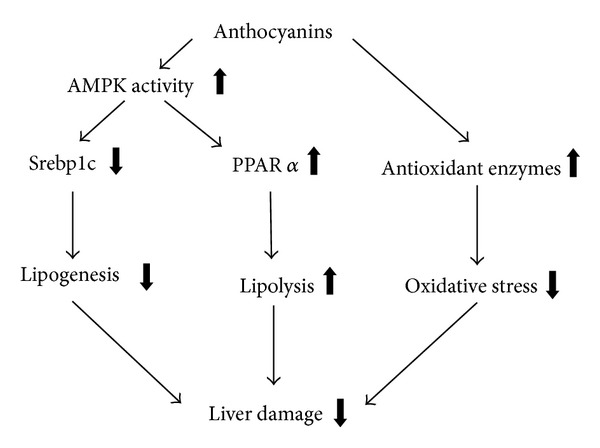
Possible mechanisms underpinning the beneficial effects of anthocyanins in NAFLD and NASH: a Srebp1c working model based on available studies. Anthocyanins may prevent the progression of liver damage related to NAFLD by three independent mechanisms: inhibition of lipogenesis by reducing Srebp1c, promotion of lipolysis by induction of PPAR*α* activity, and reduction of oxidative stress by induction of anti-oxidant enzymes. The effects of anthocyanins on lipid metabolism seem to be dependent on the activation of the AMPK pathway in hepatocytes.

A working model depicting the possible mechanisms underpinning the effects of ACN in NAFLD, based on the available findings in the literature, is presented in Figure 2. ACNs may prevent the progression of liver damage related to NAFLD by three independent mechanisms: inhibition of lipogenesis by reducing Srebp1c, promotion of lipolysis by induction of PPAR*α* activity, and reduction of oxidative stress.

On the basis of these data, it seems that ACN-rich foods can be promising for the prevention of NAFLD and its complications. Additional studies are required to clarify the molecular mechanisms and to test the specific effect of single compounds and food extracts *in vitro* and *in vivo*. Randomized controlled studies are warranted to test foods on histological damage or noninvasive biomarkers of liver damage progression in patients with NASH.

## Figures and Tables

**Table 1:** Anthocyanin concentrations in selected food sources.

Food description	Cyanidin mg/100 g	Delphinidin mg/100 g	Malvidin mg/100 g	Pelargonidin mg/100 g	Peonidin mg/100 g	Petunidin mg/100 g
*Berries *						
Arctic bramble berries (*Rubus arcticus*)	88.3			0.7		
Bilberry (*Vaccinium myrtillus*)	85.3	97.6	39.2		20.4	42.7
Blackberries (*Rubus spp.*)	99.9	0	0	0.4	0.2	0
Blueberries (*Vaccinium spp.*)						
Cultivated	8.5	35.4	67.6	0	20.3	31.5
Wild	19.4	37.6	57.2	2.6	10	23.5
Chokeberry	344.1	0.6	1.2	1	0.1	2.8
Cranberries (*Vaccinium macrocarpon*)	46.4	7.7	0.4	0	49.2	0
Currants						
Black (*Ribes nigrum*)	61.3	87.9		1.2	0.6	3.9
Red	65.5	9.3			0.2	
Golden (*Ribes aureum*)	108.8	0.7			0.1	
Elderberries (*Sambucus spp.*)	485.3	0		0		0
*Raspberries *						
Black	669			16.7	1.1	
Raspberries (*Rubus spp.*)	45.8	1.3	0.1	1	0.1	0.3
Saskatoon berries (*Amelanchier canadensis*)	110.6	50.4	10.6	0	3	6.3
Strawberries (*Fragaria X ananassa*)	1.7	0.3	0	24.8	0	0.1

*Other fruits *						
Cherries, sweet	30.2	0	0	1.4	1.5	0
Grape						
Red	1.2	2.3	39	0	3.6	2
Concord (*Vitis vinifera*)	23.8	70.6	6		4.8	14.9
Pistachio nuts, raw (*Pistacia vera*)	7.3	0	0	0	0	0
Plums						
Black diamond (with peel)	56	0	0	0	0	0
Purple	17.9				5.2	
Plums (*Prunus spp.*)	5.63	0	0	0	0.3	0

*Vegetables *						
Black beans (*P*. *vulgaris*)		18.5	10.6			15.4
Cabbage red picked	11.8					
Eggplant raw (*Solanum melongena*)		85.7				
Onions red	3.2	4.3		0	2.1	
Radicchio (*Cichorium intybus*)	127	7.7				
Radishes (*Raphanus sativus*)	0	0	0	63.1	0	0
Sweet potato purple (cooked)	10.6	0.9		0		

**Table 2:** Studies evaluating the effect of anthocyanins on hepatic lipid metabolism and hepatocellular lipotoxicity *in vitro*.

Paper	Anthocyanin	Food	Model	Effects	Mechanism
46	ACN-rich extract	Bilberry	Primary rat hepatocytes	⇓ tBH induced damage ⇓ MTT, LDH, TBARS	Antioxidant
47	ACN-rich fraction	Blueberry	HepG2 cells	⇓ OA induced TG accumulation at high doses	?
48	Anthocyanin factor	Sweet potato	HepG2 cells	⇑ pAMPK ⇓ Srepb1c, FAS	⇑ pAMPK
49	Cyanidin-3-O-*β*-glucoside	—	HepG2 cells	⇓ lipogenesis	⇑ pPKC *ζ* ⇓ MtGPAT1 translocation to OMM
50	Cyanidin chloride	Blackberry	HepG2 cells	⇑ antioxidants (SOD, catalase)	⇑ pMAPK, ⇑ Nrf2 and PPAR*α*
51	Cyanidin-3-O-*β*-glucoside	—	HepG2 cells	*⇓* ROS induced by glucose ⇑ antioxidants (GSH)	*⇑* PKA and CREB
52	Cyanidin-3-O-*β*-glucoside	—	HepG2 cells	*⇑* pAMPK and pACC, *⇑* CPT1 and FFAs oxidation	AMPK activation mediated by calmodulin kinase kinase
53	ACN-rich extract	Mulberry	HepG2 cells	*⇑* pAMPK and pACC, *⇑* PPAR*α*, CPT1 and FFAs oxidation *⇓* Srebp1c and lipogenesis	AMPK activation
54	Cyanidin	—	HepG2 cells	*⇓* lipogenesis *⇑* lipolysis	PPAR*αβ*/*δ* agonist

AMPK: adenosine monophosphate protein kinase; Srebp1c: sterol regulated element binding protein 1c; ACC: acetyl-coenzyme A carboxylase; p: phospho; glycerol 3 phophate acyl transferase; PKC: protein kinase C; OMM: outer mitochondrial membrane; SOD: superoxide dismutase; MAPK: mitogen associated protein kinase; Nrf2: nuclear factor erythroid 2-related factor 2; PPAR*α*: *β*/*δ* peroxisomes proliferator activated receptor *α*; ROS: reactive oxygen species; GSH: reduced glutathione; PKA: protein kinase A; CREB: cAMP-response element binding protein; CPT-1: carnitine-palmytoil-transferase-1; ACN: anthocyanins; OA: oleic acid; tBH: tert-butyl hydroperoxide; MTT: 3-(4,5-dimethyl-2-thiazolyl)-2,5-diphenyl-2H-tetrazolium bromide; LDH: lactate dehydrogenase; TBARS: thiobarbituric acid reacting substances.

**Table 3:** Studies evaluating the effect of anthocyanins on hepatic steatosis and steatohepatitis *in vivo*.

Paper	Anthocyanin	Food	Model	Metabolic effects	Molecular effects
48	Anthocyanin factor	Sweet potato	Mice fed HFD	⇓ weight gain *⇓* steatosis	*⇑* pAMPK and pACC *⇓* Srepb1c, FAS, ACC
49	Cyaniding-3-O-*β*-glucoside	—	KKAy mice	*⇓* steatosis	*⇓* GPAT1 translocation to OMM
51	Cyanidin-3-O-*β*-glucoside	—	db/db mice	*⇑* antioxidants (GSH) *⇓* steatosis, ROS, and inflammation	*⇑* PKA and CREB
55	Cyanidin-3-O-*β*-glucoside	Blackcurrant	Rats	*⇓* steatosis *⇓* hepatic saturated FAs *⇑* antioxidants	?
56	Cyanidin-3-O-*β*-glucoside	—	C57Bl/6 on HFD and db/db	*⇓* glucose and IR *⇓* cytokines and adipose tissue inflammation *⇓* steatosis	*⇓* hepatic JNK *⇓* hepatic FOXO1 activity and gluconeogenesis
57	Several	Tart cherry	Dahl Salt-Sensitive rat	*⇓* fasting glucose *⇓* hyperlipidemia *⇓* hyperinsulinemia *⇓* steatosis	*⇑* PPAR*α* *⇑* acyl-coenzyme A oxidase
58	—	Vitis coignetiae Pulliat leaves (yama-budo)	Rats on HFD choline deficient diet	*⇓* liver enzymes and liver fibrosis *⇓* CYP2E1 *⇑* antioxidants	?
59	Several	Moro orange juice	C57Bl/6 mice on HFD	*⇓* weight gain *⇓* IR, *⇓* TGs, *⇓* steatosis	*⇓* LXR, FAS *⇑* PPAR*α*, Srebp1c
27	Several	Wild blueberry (Vaccinium angustifolium)	Zucker rats	*⇓* hyperlipidemia	*⇑* PPAR*α* *⇓* Srebp1c
60	—	Blueberry	Zucker rats on HFD	*⇓* IR and lipids *⇓* adiposity *⇓* steatosis	*⇑* PPAR*α*
61	—	Mulberry	Hamsters on HFD	*⇓* weight gain and visceral fat, *⇓* TGs, chol, FFAs *⇓* steatosis	*⇓* HMG-CoA, FAS *⇑* PPAR*α*, CPT-1
62	Several	Elderberry	Hamsters fed high fat fish oil	*⇓* serum lipids *⇓* steatosis *⇓* lipoperoxidation	?
63	—	Mulberry	Rats on HFD	*⇓* serum lipids *⇓* hepatic and serum lipoperoxidation	*⇑* antioxidants

HFD: high fat diet; IR: insulin resistance; TGs: triglycerides; LXR: liver X receptor; FAS: fatty acid synthase; GAPT1: glycerol 3 phosphate acyl transferase; PPAR*α*: peroxisomes proliferator activated receptor *α*; chol: cholesterol; FFAs: free fatty acids; CPT-1: carnitine-palmitoyl-transferase-1; HMG-CoA red: 3-hydroxymethyl-3-glutaryl-coenzyme A reductase; p: phospho; AMPK: adenosine monophoshopate protein kinase; Srebp1c: sterol regulated element binding protein 1c; ACC: acetyl-coenzyme A carboxylase; ROS: reactive oxygen species; JNK: c-Jun N-terminal kinase; FOXO1: forkhead box O1.

**Table 4:** Studies evaluating the effect of anthocyanins on hepatic steatosis and steatohepatitis in patients.

Paper	Anthocyanin	Food	Subjects	Metabolic effects	Mechanism
64	Acylated anthocyanins	Purple sweet potato beverage 8 wks	Healthy humans with borderline hepatitis	⇓ GGT (AST, ALT) ⇓ oxidative stress	⇓ oxidative stress

GGT: g-glutamyl transferase; ALT: alanine aminotransferase; AST: aspartate aminotransferase.

## References

[1] Mensah GA, Mokdad AH, Ford E (2004). Obesity, metabolic syndrome, and type 2 diabetes: emerging epidemics and their cardiovascular implications. *Cardiology Clinics*.

[2] Blachier M, Leleu H, Peck-Radosavljevic M, Valla DC, Roudot-Thoraval F (2013). The burden of liver disease in Europe: a review of available epidemiological data. *Journal of Hepatology*.

[3] Guerrero R, Vega GL, Grundy SM, Browning JD (2009). Ethnic differences in hepatic steatosis: an insulin resistance paradox?. *Hepatology*.

[4] WHO (2011). *Global Status Report on Non Communicable Diseases 2010*.

[5] Cecchini M, Sassi F, Lauer JA, Lee YY, Guajardo-Barron V, Chisholm D (2010). Tackling of unhealthy diets, physical inactivity, and obesity: health effects and cost-effectiveness. *The Lancet*.

[6] Traka MH, Mithen RF (2011). Plant science and human nutrition: challenges in assessing health-promoting properties of phytochemicals. *The Plant Cell*.

[7] Bueno JM, Sáez-Plaza P, Ramos-Escudero F, Jiménez AM, Fett R, Asuero AG (2012). Analysis and antioxidant capacity of anthocyanin pigments. Part II: chemical structure, color, and intake of anthocyanins. *Critical Reviews in Analytical Chemistry*.

[8] Castaneda-Ovando A, de Lourdes Pacheco-Hernandez M, Paez-Hernandez ME, Rodriguez C JA, Galan-Vidal A (2009). Chemical studies of anthocyanins: a review. *Food Chemistry*.

[9] Kühnau J (1976). The flavonoids. A class of semi-essential food components: their role in human nutrition. *Nutrition, Metabolism and Cardiovascular Disease*.

[10] Hertog MGL, Hollman PCH, Katan MB, Kromhout D (1993). Intake of potentially anticarcinogenic flavonoids and their determinants in adults in The Netherlands. *Nutrition and Cancer*.

[11] Wu X, Beecher GR, Holden JM, Haytowitz DB, Gebhardt SE, Prior RL (2006). Concentrations of anthocyanins in common foods in the United States and estimation of normal consumption. *Journal of Agricultural and Food Chemistry*.

[12] Yang M, Koo SI, Song WO, Chun OK (2011). Food matrix affecting anthocyanin bioavailability: review. *Current Medicinal Chemistry*.

[13] Wang L-S, Stoner GD (2008). Anthocyanins and their role in cancer prevention. *Cancer Letters*.

[14] del Rio D, Borges G, Crozier A (2010). Berry flavonoids and phenolics: bioavailability and evidence of protective effects. *British Journal of Nutrition*.

[15] Chong MF-F, MacDonald R, Lovegrove JA (2010). Fruit polyphenols and CVD risk: a review of human intervention studies. *British Journal of Nutrition*.

[16] Kong J-M, Chia L-S, Goh N-K, Chia T-F, Brouillard R (2003). Analysis and biological activities of anthocyanins. *Phytochemistry*.

[17] Galvano F, La Fauci L, Lazzarino G (2004). Cyanidins: metabolism and biological properties. *Journal of Nutritional Biochemistry*.

[18] Zafra-Stone S, Yasmin T, Bagchi M, Chatterjee A, Vinson JA, Bagchi D (2007). Berry anthocyanins as novel antioxidants in human health and disease prevention. *Molecular Nutrition and Food Research*.

[19] Prior RL, Wu X (2006). Anthocyanins: structural characteristics that result in unique metabolic patterns and biological activities. *Free Radical Research*.

[20] Sasaki R, Nishimura N, Hoshino H (2007). Cyanidin 3-glucoside ameliorates hyperglycemia and insulin sensitivity due to downregulation of retinol binding protein 4 expression in diabetic mice. *Biochemical Pharmacology*.

[21] Basu A, Du M, Leyva MJ (2010). Blueberries decrease cardiovascular risk factors in obese men and women with metabolic syndrome. *Journal of Nutrition*.

[22] del Bo’ C, Kristo AS, Kalea AZ (2012). The temporal effect of a wild blueberry (*Vaccinium angustifolium*)-enriched diet on vasomotor tone in the Sprague-Dawley rat. *Nutrition, Metabolism and Cardiovascular Diseases*.

[23] Vendrame S, Daugherty A, Kristo AS, Riso P, Klimis-Zacas D (2013). Wild blueberry (*Vaccinium angustifolium*) consumption improves inflammatory status in the obese Zucker rat model of the metabolic syndrome. *Journal of Nutritional Biochemistry*.

[24] Kristo AS, Kalea AZ, Schuschke DA, Klimis-Zacas D (2013). Attenuation of alpha-adrenergic-induced vasoconstriction by dietary wild blueberries (*Vaccinium angustifolium*) is mediated by the NO-cGMP pathway in spontaneously hypertensive rats (SHRs). *International Journal of Food Science and Nutrition*.

[25] Tsuda T, Horio F, Uchida K, Aoki H, Osawa T (2003). Dietary cyanidin 3-O-*β*-D-glucoside-rich purple corn color prevents obesity and ameliorates hyperglycemia in mice. *Journal of Nutrition*.

[26] Titta L, Trinei M, Stendardo M (2010). Blood orange juice inhibits fat accumulation in mice. *International Journal of Obesity*.

[27] Vendrame S, Daugherty A, Kristo AS, Klimis-Zacas D (2013). Wild blueberry (*Vaccinium angustifolium*)-enriched diet improves dyslipidaemia and modulates the expression of genes related to lipid metabolism in obese Zucker rats. *British Journal of Nutrition*.

[28] Marchesini G, Brizi M, Blanchi G (2001). Nonalcoholic fatty liver disease: a feature of the metabolic syndrome. *Diabetes*.

[29] Day CP (2006). From fat to inflammation. *Gastroenterology*.

[30] Kleiner DE, Brunt EM, van Natta M (2005). Design and validation of a histological scoring system for nonalcoholic fatty liver disease. *Hepatology*.

[31] Bugianesi E, Leone N, Vanni E (2002). Expanding the natural history of nonalcoholic steatohepatitis: from cryptogenic cirrhosis to hepatocellular carcinoma. *Gastroenterology*.

[32] Browning JD, Szczepaniak LS, Dobbins R (2004). Prevalence of hepatic steatosis in an urban population in the United States: impact of ethnicity. *Hepatology*.

[33] Bellentani S, Saccoccio G, Masutti F (2000). Prevalence of and risk factors for hepatic steatosis in northern Italy. *Annals of Internal Medicine*.

[34] Cohen JC, Horton JD, Hobbs HH (2011). Human fatty liver disease: old questions and new insights. *Science*.

[35] Yamaguchi K, Yang L, McCall S (2007). Inhibiting triglyceride synthesis improves hepatic steatosis but exacerbates liver damage and fibrosis in obese mice with nonalcoholic steatohepatitis. *Hepatology*.

[36] Donnelly KL, Smith CI, Schwarzenberg SJ, Jessurun J, Boldt MD, Parks EJ (2005). Sources of fatty acids stored in liver and secreted via lipoproteins in patients with nonalcoholic fatty liver disease. *Journal of Clinical Investigation*.

[37] Bugianesi E, Gastaldelli A, Vanni E (2005). Insulin resistance in non-diabetic patients with non-alcoholic fatty liver disease: sites and mechanisms. *Diabetologia*.

[38] Marchesini G, Brizi M, Morselli-Labate AM (1999). Association of nonalcoholic fatty liver disease with insulin resistance. *American Journal of Medicine*.

[39] Fabbrini E, Mohammed BS, Magkos F, Korenblat KM, Patterson BW, Klein S (2008). Alterations in adipose tissue and hepatic lipid kinetics in obese men and women with nonalcoholic fatty liver disease. *Gastroenterology*.

[40] Rametta R, Mozzi E, Dongiovanni P (2013). Increased insulin receptor substrate 2 expression is associated with steatohepatitis and altered lipid metabolism in obese subjects. *International Journal of Obesity*.

[41] Day CP, James OFW (1998). Steatohepatitis: a tale of two “hits”?. *Gastroenterology*.

[42] Valenti L, Fracanzani AL, Fargion S (2009). The immunopathogenesis of alcoholic and nonalcoholic steatohepatitis: two triggers for one disease?. *Seminars in Immunopathology*.

[43] Miele L, Valenza V, La Torre G (2009). Increased intestinal permeability and tight junction alterations in nonalcoholic fatty liver disease. *Hepatology*.

[44] Bardella MT, Valenti L, Pagliari C (2004). Searching for coeliac disease in patients with non-alcoholic fatty liver disease. *Digestive and Liver Disease*.

[45] Dongiovanni P, Anstee QM, Valenti L (2013). Genetic predisposition in NAFLD and NASH: impact on severity of liver disease and response to treatment. *Current Pharmaceutical Design*.

[46] Valenti L, Alisi A, Nobili V (2012). I148M *PNPLA3* variant and progressive liver disease: a new paradigm in hepatology. *Hepatology*.

[47] Valentová K, Ulrichová J, Cvak L, Šimánek V (2006). Cytoprotective effect of a bilberry extract against oxidative damage of rat hepatocytes. *Food Chemistry*.

[48] Liu Y, Wang D, Zhang D (2011). Inhibitory effect of blueberry polyphenolic compounds on oleic acid-induced hepatic steatosis in vitro. *Journal of Agricultural and Food Chemistry*.

[49] Hwang YP, Choi JH, Han EH (2011). Purple sweet potato anthocyanins attenuate hepatic lipid accumulation through activating adenosine monophosphate-activated protein kinase in human HepG2 cells and obese mice. *Nutrition Research*.

[50] Guo H, Li D, Ling W, Feng X, Xia M (2011). Anthocyanin inhibits high glucose-induced hepatic mtGPAT1 activation and prevents fatty acid synthesis through PKC *ζ*. *Journal of Lipid Research*.

[51] Cho BO, Ryu HW, Jin CH (2011). Blackberry extract attenuates oxidative stress through up-regulation of Nrf2-dependent antioxidant enzymes in carbon tetrachloride-treated rats. *Journal of Agricultural and Food Chemistry*.

[52] Zhu W, Jia Q, Wang Y, Zhang Y, Xia M (2012). The anthocyanin cyanidin-3-O-*β*-glucoside, a flavonoid, increases hepatic glutathione synthesis and protects hepatocytes against reactive oxygen species during hyperglycemia: involvement of a cAMP-PKA-dependent signaling pathway. *Free Radical Biology and Medicine*.

[53] Guo H, Liu G, Zhong R, Wang Y, Wang D, Xia M (2012). Cyanidin-3-*O*-*β*-glucoside regulates fatty acid metabolism via an AMP-activated protein kinase-dependent signaling pathway in human HepG2 cells. *Lipids in Health and Disease*.

[54] Chang JJ, Hsu MJ, Huang HP (2013). Mulberry anthocyanins inhibit oleic acid induced lipid accumulation by reduction of lipogenesis and promotion of hepatic lipid clearance. *Journal of Agricultural and Food Chemistry*.

[55] Jia Y, Kim JY, Jun HJ (2013). Cyanidin is an agonistic ligand for peroxisome proliferator-activated receptor-alpha reducing hepatic lipid. *Biochimica et Biophysica Acta*.

[56] Frank J, Kamal-Eldin A, Lundh T, Määttä K, Törrönen R, Vessby B (2002). Effects of dietary anthocyanins on tocopherols and lipids in rats. *Journal of Agricultural and Food Chemistry*.

[57] Guo H, Xia M, Zou T, Ling W, Zhong R, Zhang W (2012). Cyanidin 3-glucoside attenuates obesity-associated insulin resistance and hepatic steatosis in high-fat diet-fed and db/db mice via the transcription factor FoxO1. *Journal of Nutritional Biochemistry*.

[58] Seymour EM, Singer AAM, Kirakosyan A, Urcuyo-Llanes DE, Kaufman PB, Bolling SF (2008). Altered hyperlipidemia, hepatic steatosis, and hepatic peroxisome proliferator-activated receptors in rats with intake of tart cherry. *Journal of Medicinal Food*.

[59] Takayama F, Nakamoto K, Kawasaki H (2009). Beneficial effects of Vitis coignetiae Pulliat leaves on nonalcoholic steatohepatitis in a rat model. *Acta Medica Okayama*.

[60] Salamone F, Li Volti G, Titta L (2012). Moro orange juice prevents fatty liver in mice. *World Journal of Gastroenterology*.

[61] Seymour EM, Tanone II, Urcuyo-Llanes DE (2011). Blueberry intake alters skeletal muscle and adipose tissue peroxisome proliferator-activated receptor activity and reduces insulin resistance in obese rats. *Journal of Medicinal Food*.

[62] Peng C-H, Liu L-K, Chuang C-M, Chyau C-C, Huang C-N, Wang C-J (2011). Mulberry water extracts possess an anti-obesity effect and ability to inhibit hepatic lipogenesis and promote lipolysis. *Journal of Agricultural and Food Chemistry*.

[63] Dubey P, Jayasooriya AP, Cheema SK (2012). Fish oil induced hyperlipidemia and oxidative stress in BioF1B hamsters is attenuated by elderberry extract. *Applied Physiology, Nutrition and Metabolism*.

[64] Yang X, Yang L, Zheng H (2010). Hypolipidemic and antioxidant effects of mulberry (*Morus alba* L.) fruit in hyperlipidaemia rats. *Food and Chemical Toxicology*.

[65] Takikawa M, Inoue S, Horio F, Tsuda T (2010). Dietary anthocyanin-rich bilberry extract ameliorates hyperglycemia and insulin sensitivity via activation of amp-activated protein kinase in diabetic mice. *Journal of Nutrition*.

[66] Suda I, Ishikawa F, Hatakeyama M (2008). Intake of purple sweet potato beverage affects on serum hepatic biomarker levels of healthy adult men with borderline hepatitis. *European Journal of Clinical Nutrition*.

[67] Nobili V, Bedogni G, Alisi A (2011). Docosahexaenoic acid supplementation decreases liver fat content in children with non-alcoholic fatty liver disease: double-blind randomised controlled clinical trial. *Archives of Disease in Childhood*.

